# Supplementary material for: Direct visualization of a molecular handshake that governs kin recognition and tissue formation in myxobacteria
Source: Nat Commun. 2019 Jul 12;10:3073. doi: 10.1038/s41467-019-11108-w (PMC6626042; doi:10.1038/s41467-019-11108-w)
Supplement: Supplementary file 1 — Supplementary information [file 41467_2019_11108_MOESM1_ESM.pdf]

Supplementary Information

Title: Direct visualization of a molecular handshake that governs kin recognition and tissue formation in myxobacteria

Authors: Pengbo Cao and Daniel Wall*

Affiliations: Department of Molecular Biology, University of Wyoming, 1000 E University Avenue, Laramie, WY, 82071, USA

***Correspondence to:** dwall2@uwyo.edu

Supplementary Figures

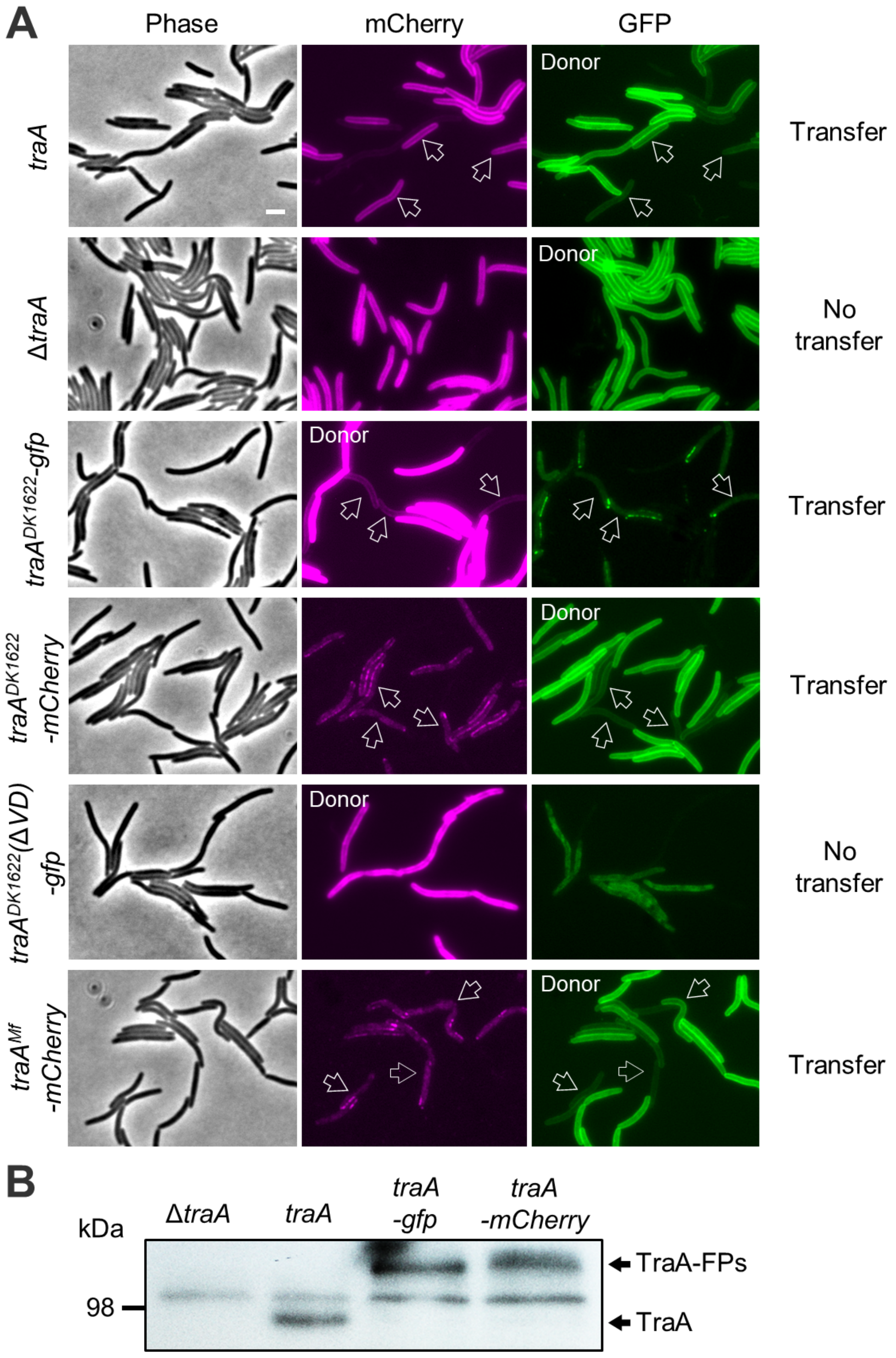

Supplementary Figure 1. Functional characterization of TraA-FPs used in this study. (**A**) Transfer assay testing the function of engineered *traA* alleles. Representative images are shown. Donor strains expressing either SS_{OM}-GFP or SS_{OM}-mCherry as transfer cargo are indicated. Except for the positive control, recipients are Δ*traA* strains expressing indicated *traA* alleles at the Mx8 attachment site (labeled on the left). Arrows highlight recipients that acquired fluorescent cargo. (**B**) Western blot analyzing TraA-FPs with anti-TraA serum. See **Supplementary Table 1** for strain details. Scale bar = 1 μm. Source data are provided as a Source Data file.

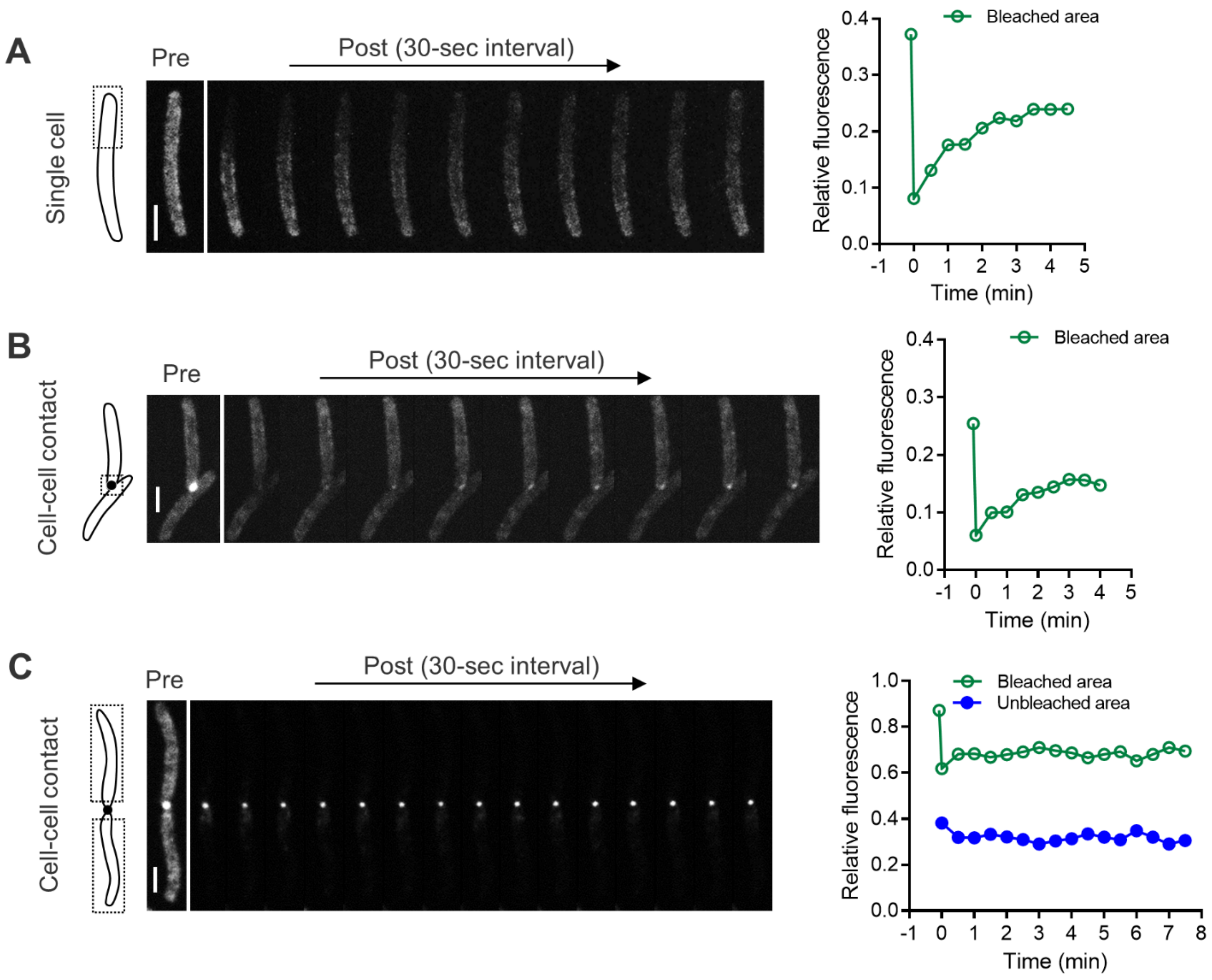

Supplementary Figure 2. FRAP analyses of TraA-GFP fluidity. (**A-C**) Additional examples of TraA-GFP FRAP analyses. Bleached areas are indicated with dashed borders. Relative fluorescence measures the ratio of fluorescence intensity of the bleached areas to that of the whole cells before and after photobleaching. Scale bar = 1 μm. Source data are provided as a Source Data file.

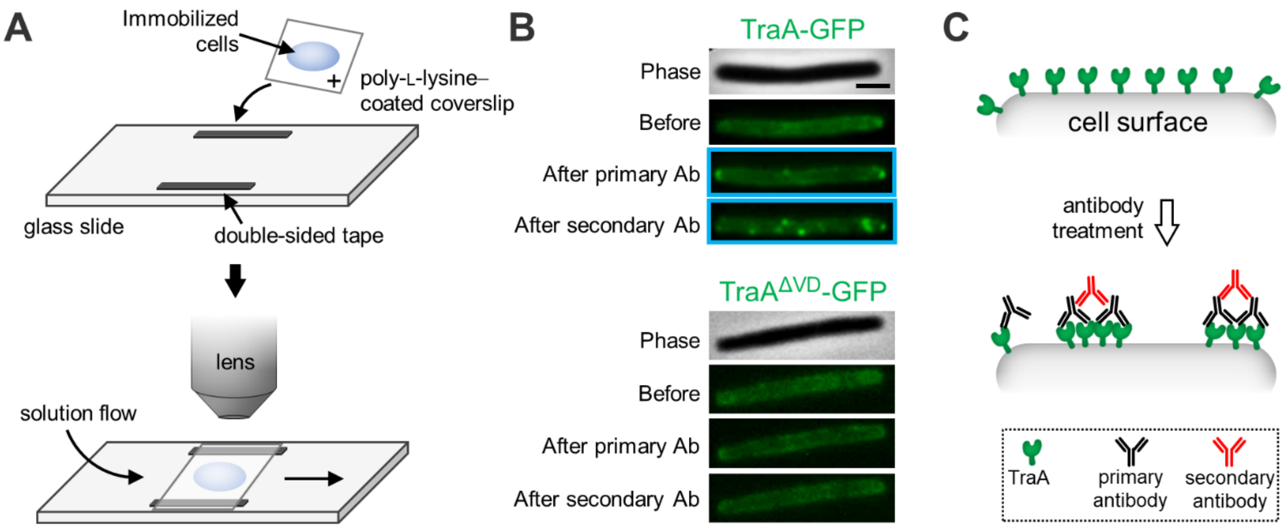

Supplementary Figure 3. TraA clusters induced by multivalent binding of antibodies. (**A**) Schematic of the custom flow cell system. See Methods for details. (**B**) Representative images of antibody treatments on TraA-GFP cells (n = 41) and TraA$^{\Delta VD}$-GFP cells (n = 33). Incubation with primary antibodies that target the VD in TraA and a secondary antibody induce the formation of TraA-GFP foci (blue borders). TraA$^{\Delta VD}$-GFP is a negative control. Scale bar = 1 μm. (**C**) Schematic for how multivalent antibodies induce TraA clusters shown in **B**.

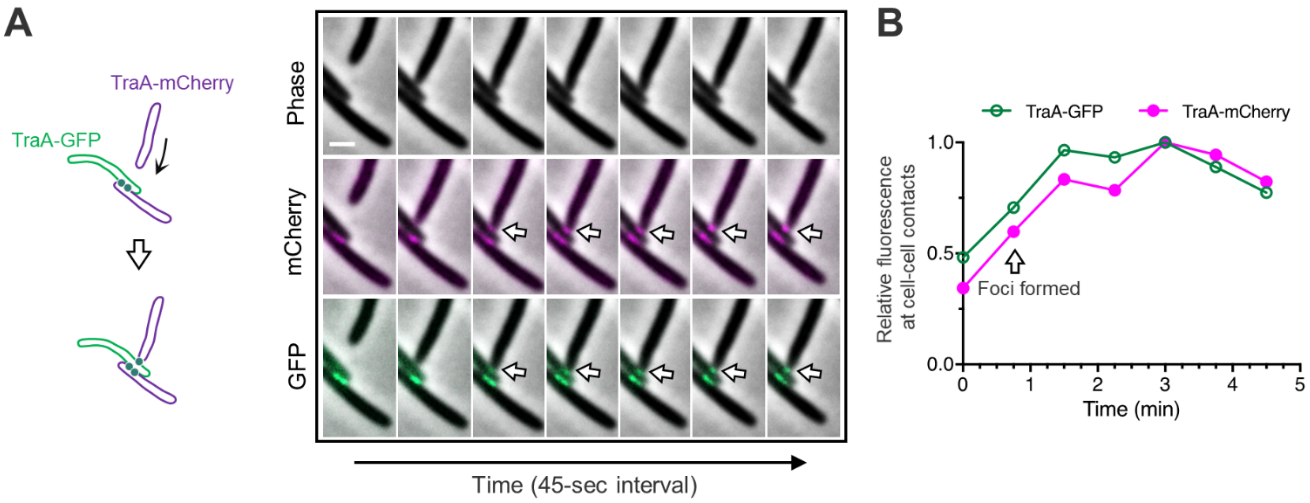

Supplementary Figure 4. TraA proteins from opposing cells form clusters simultaneously upon physical contact. (**A**) Representative time-lapse series showing TraA-mCherry cells encountering a TraA-GFP cell. A cartoon illustration is shown on the left. Arrows in the images indicate foci that form at the contact interface. Scale bar = 1 µm. (**B**) Fluorescence profiles of TraA-mCherry and TraA-GFP at the cell-cell contact point, starting from the second frame of the time series shown in (**A**) when physical contact was initiated. Source data are provided as a Source Data file.

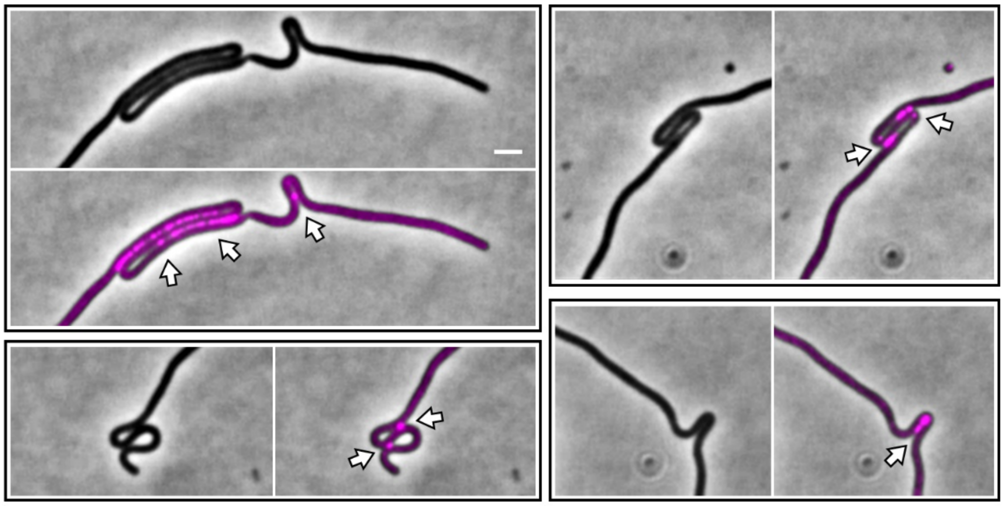

Supplementary Figure 5. Self-recognition and adhesion by single filamentous cells. TraA-mCherry cells were treated with cephalexin. Phase contrast and merged with fluorescent images shown. Foci highlighted with arrows. Scale bar = 1 μm.

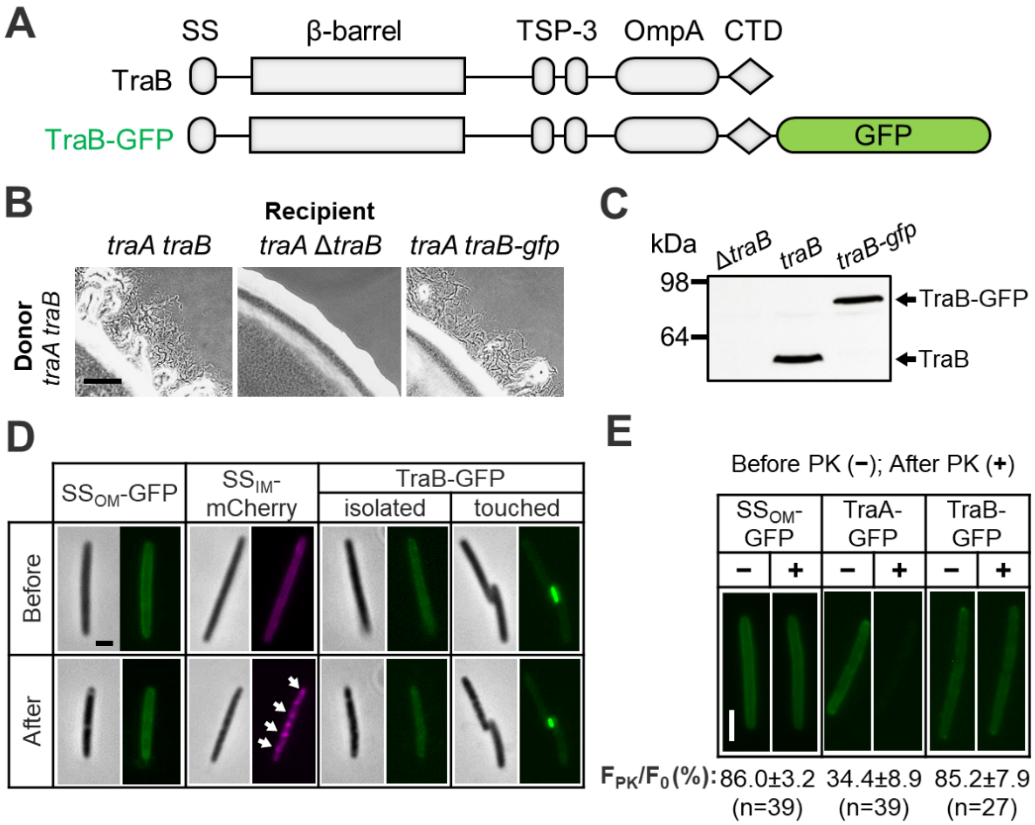

Supplementary Figure 6. Construction and characterization of TraB-GFP. (**A**) Domain architecture of TraB and TraB-GFP. TSP-3, thrombospondin type 3 (Pfam02412); CTD, carboxyl terminal domain. (**B**) A stimulation (extracellular complementation) assay demonstrating the function of TraB-GFP. This assay tests the transfer of OM lipoproteins to recipients and the rescue of a motility defect by OME[1]. Expression of *traB-gfp* in a Δ*traB* donor restored OME, as indicated by the emergent flares from colony edges (motility rescue). A Δ*traB* donor and a donor harboring native *traB* were used as negative and positive controls. Scale bar = 200 µm. (**C**) Immunoblot confirming the construction of TraB-GFP with anti-TraB serum. (**D**) Plasmolysis assay reveals the OM localization of TraB-GFP (right). Representative phase contrast and fluorescent images showing single cells before and after treatments with 0.5 M NaCl solution. SS_{OM}-GFP and SS_{IM}-mCherry served as controls (left). Arrows highlight the collapse of the inner membrane following plasmolysis as indicated by the SS_{IM}-mCherry reporter. OM proteins

retained envelope localization after plasmolysis. Scale bar = 0.5 μm. (**E**) PK accessibility assay on TraB. Representative images of live cells before (−) and after (+) PK treatment are shown. SS_{OM}-GFP (inaccessible by PK) and TraA-GFP (accessible by PK) served as controls. F_{PK}, fluorescence intensity of whole cells after PK treatment; F_0, fluorescence intensity before PK treatment. Scale bar = 1 μm. See **Supplementary Table 1** for strain details. Source data are provided as a Source Data file.

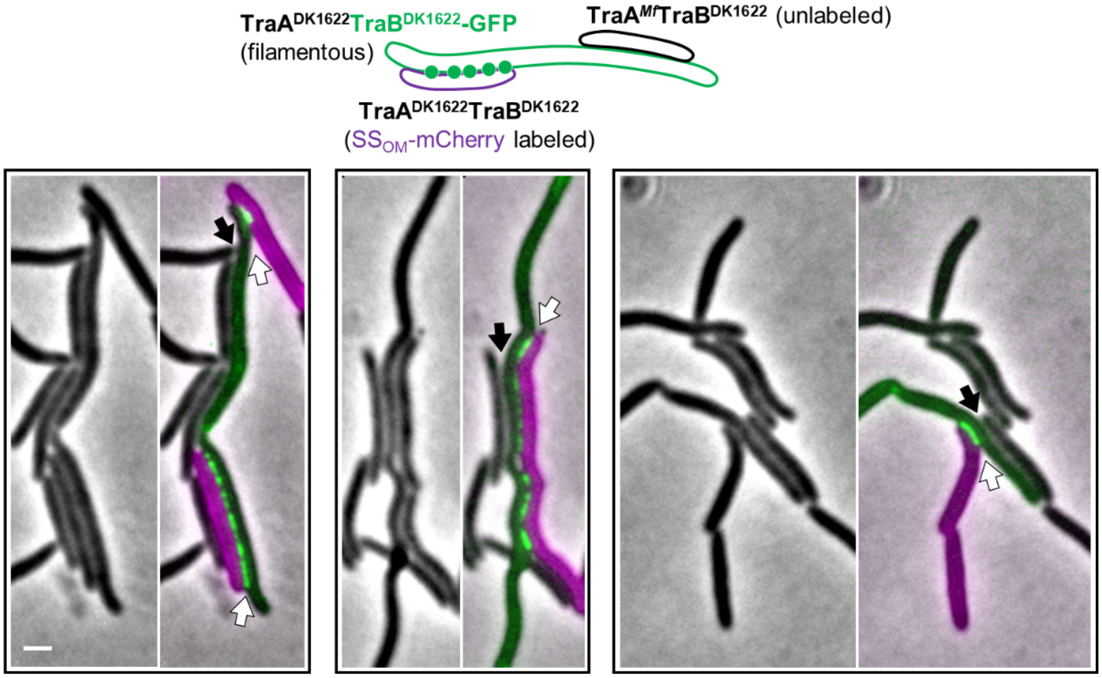

Supplementary Figure 7. TraB clusters at the interface between cells bearing compatible TraA receptors. Mixing of three differently labeled strains expressing different TraA receptors but the same TraB reporter (illustrated above). TraB^{DK1622}-GFP cells were treated with cephalexin to induce filamentation and increase the likelihood of single cells adhering with two other strains. White arrows indicate the interfaces between TraA^{DK1622} TraB^{DK1622}-GFP and TraA^{DK1622} TraB^{DK1622} cells (mCherry labeled); black arrows indicate the interfaces between TraA^{DK1622} TraB^{DK1622}-GFP and TraA^{Mf} TraB^{DK1622} cells (unlabeled). See **Supplementary Table 1** for strain details. Scale bar = 1 µm.

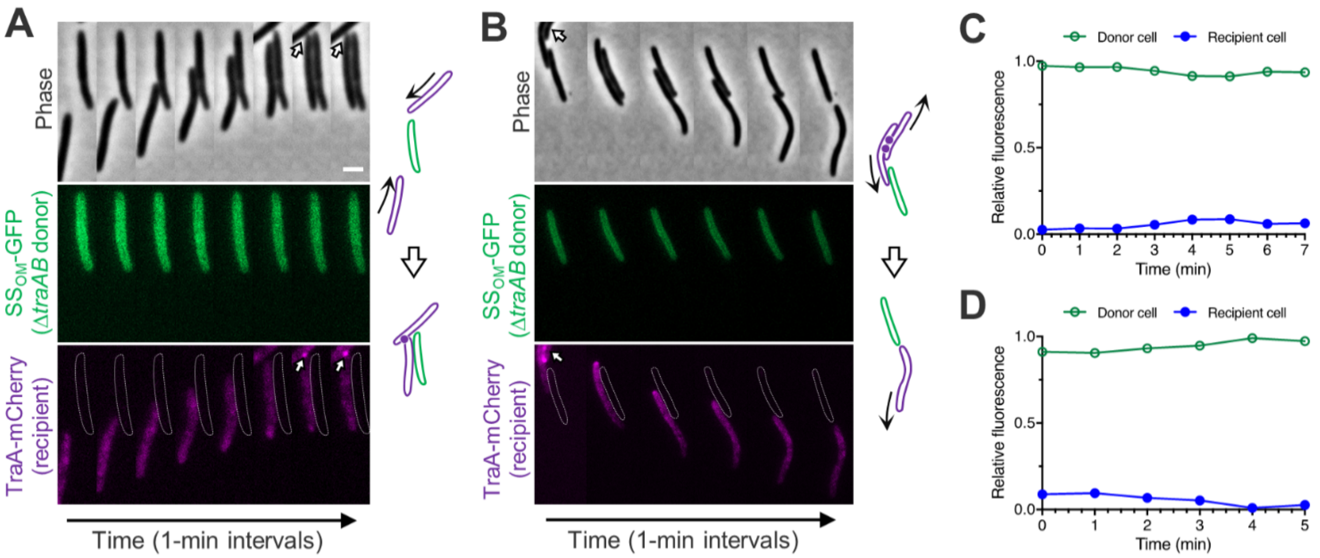

Supplementary Figure 8. TraA clustering and cargo transfer require the interaction between two *traAB*⁺ cells. (**A**-**B**) Negative controls for the time-lapse series shown in **Fig. 5A**. (**A**) A TraA-mCherry recipient first encountered a Δ*traAB* donor (outlined bottom panel; no foci formed, no cargo transfer) and then interacted with another TraA-mCherry cell (foci formed). (**B**) Arrows show the interaction between two TraA-mCherry cells (foci formed), the lower of which then encountered a Δ*traAB* donor (outlined bottom panel; foci dissolved, no transfer). The SS_{OM}-GFP fluorescence intensities of the donors and TraA-mCherry recipients in (**A**) and (**B**) were quantitatively analyzed in (**C**) and (**D**), respectively. Scale bar = 1 μm. Source data are provided as a Source Data file.

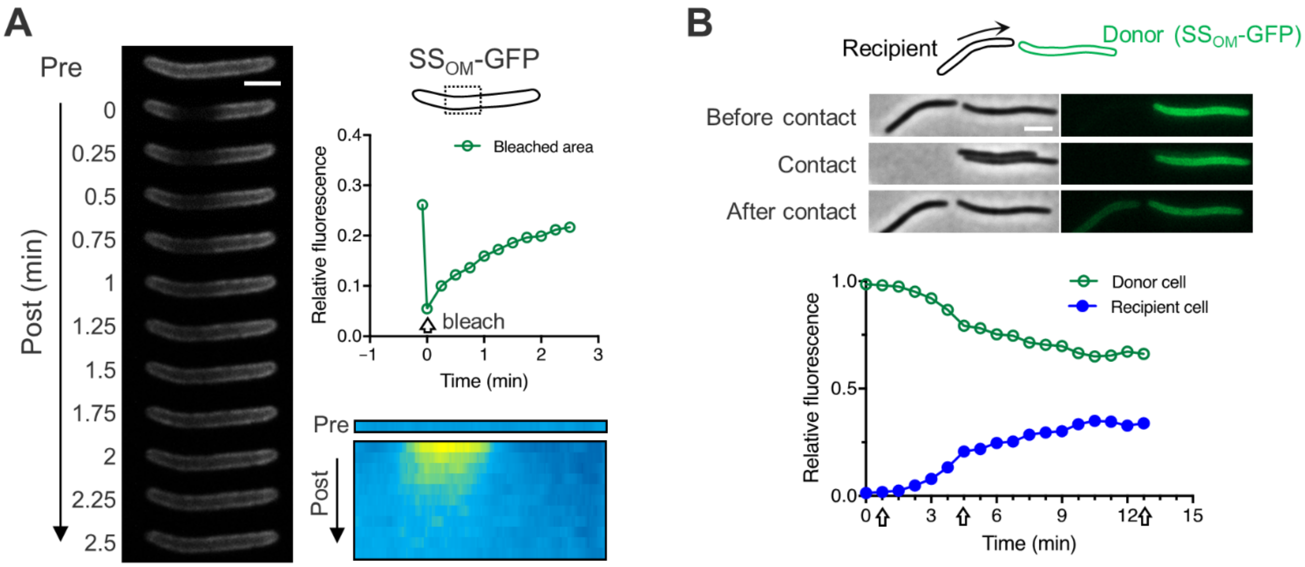

Supplementary Figure 9. OM cargo is fluid and transfers efficiently. (**A**) FRAP analysis of SS_{OM}-GFP fluidity in the OM. Relative fluorescence represents the ratio of fluorescence intensity of the bleached area (dashed borders) to that of the whole cell. SS_{OM}-GFP fluorescence intensity profiles along the analyzed cell before and after photobleaching are shown as kymograph. Blue, high intensity; yellow, low intensity. The diffusion coefficient of SS_{OM}-GFP is 0.010 ± 0.002 μm^2 s^{-1}. (**B**) Quantitative analysis of cargo transfer during the time series shown in Fig. 5A. Phase contrast and fluorescent micrographs from three time points shown (top). Arrows along x-axis in the graph bracket the time window of transfer shown above. Scale bar = 1 µm. Source data are provided as a Source Data file.

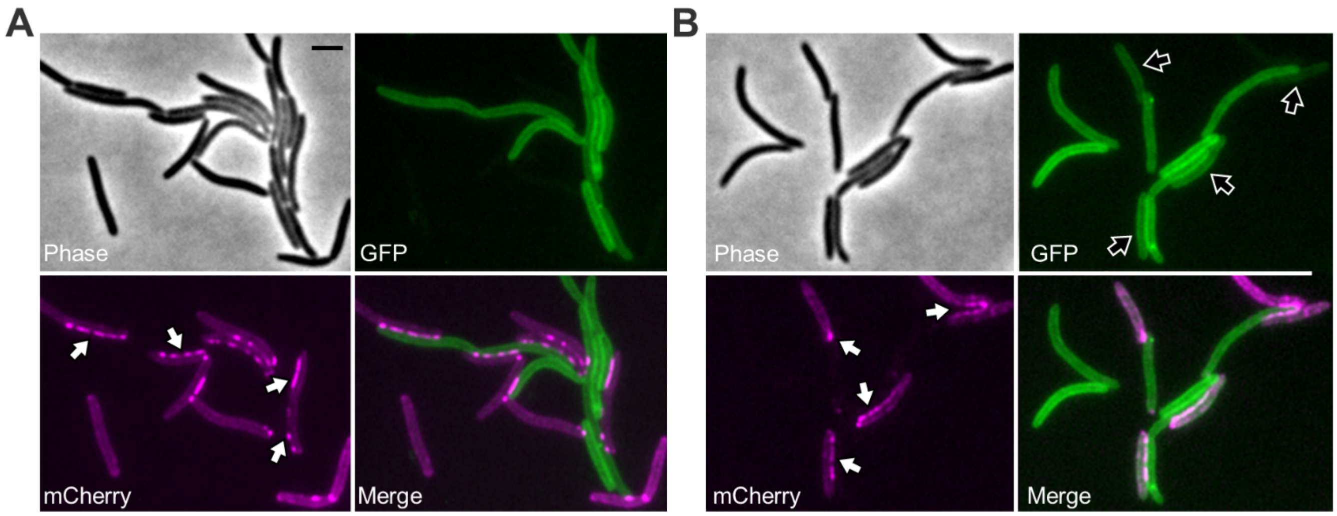

Supplementary Figure 10. Cell motility is not required for TraA cluster formation but is required for cargo transfer. (**A**) Representative images of nonmotile donors harboring SS_{OM}-GFP mixed with nonmotile TraA-mCherry recipients. White arrows indicate foci. (**B**) Representative images of the same nonmotile donors mixed with motile TraA-mCherry recipients. White arrows indicate TraA-mCherry foci; black arrows highlight recipients that acquired SS_{OM}-GFP. See **Supplementary Table 1** for strain details. Scale bar = 1 μm.

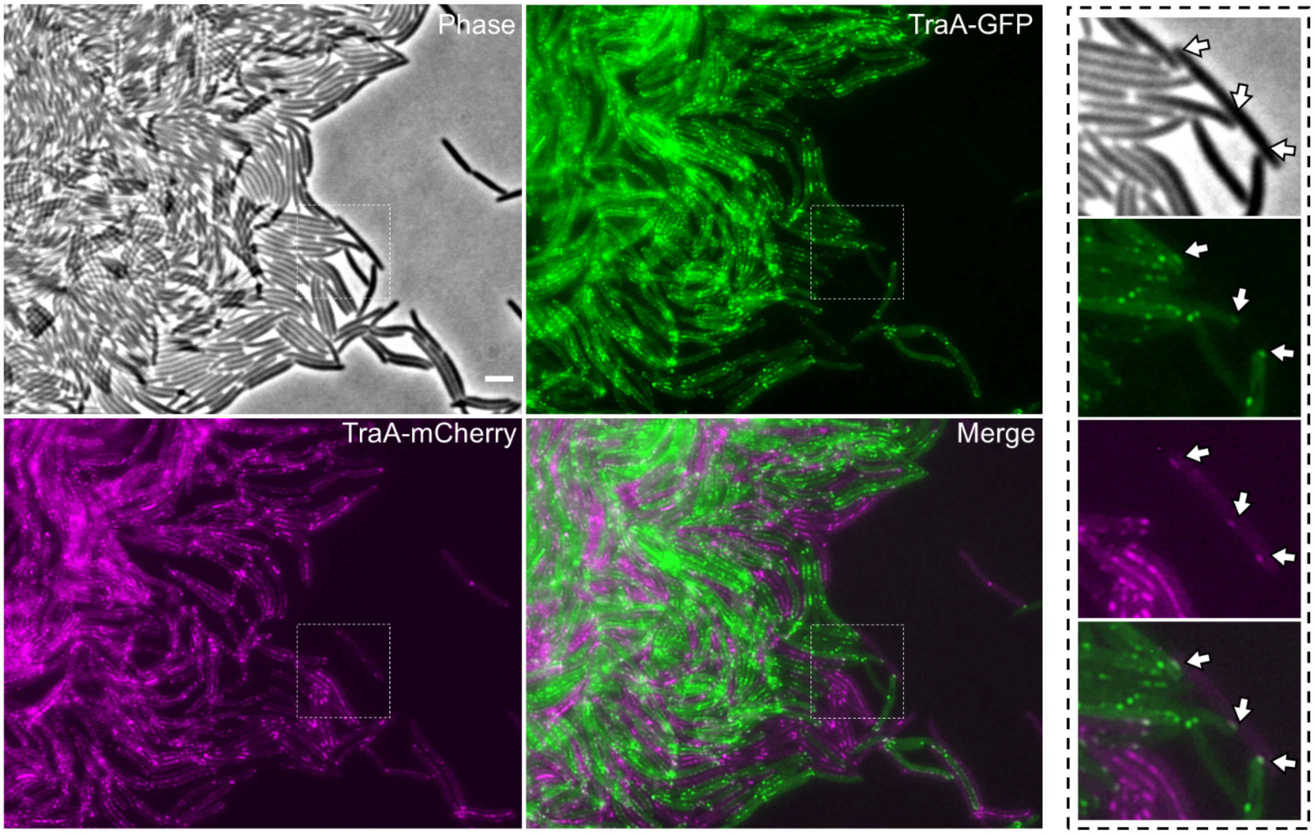

Supplementary Figure 11. TraA/B recognition results in cell-cell junctions and tissue-like structures. TraA-GFP cells were co-incubated with TraA-mCherry cells at a high density. Dashed borders on the left are enlarged in the right column to show colocalization between TraA-GFP and TraA-mCherry foci (arrows). Scale bar = 2 μm.

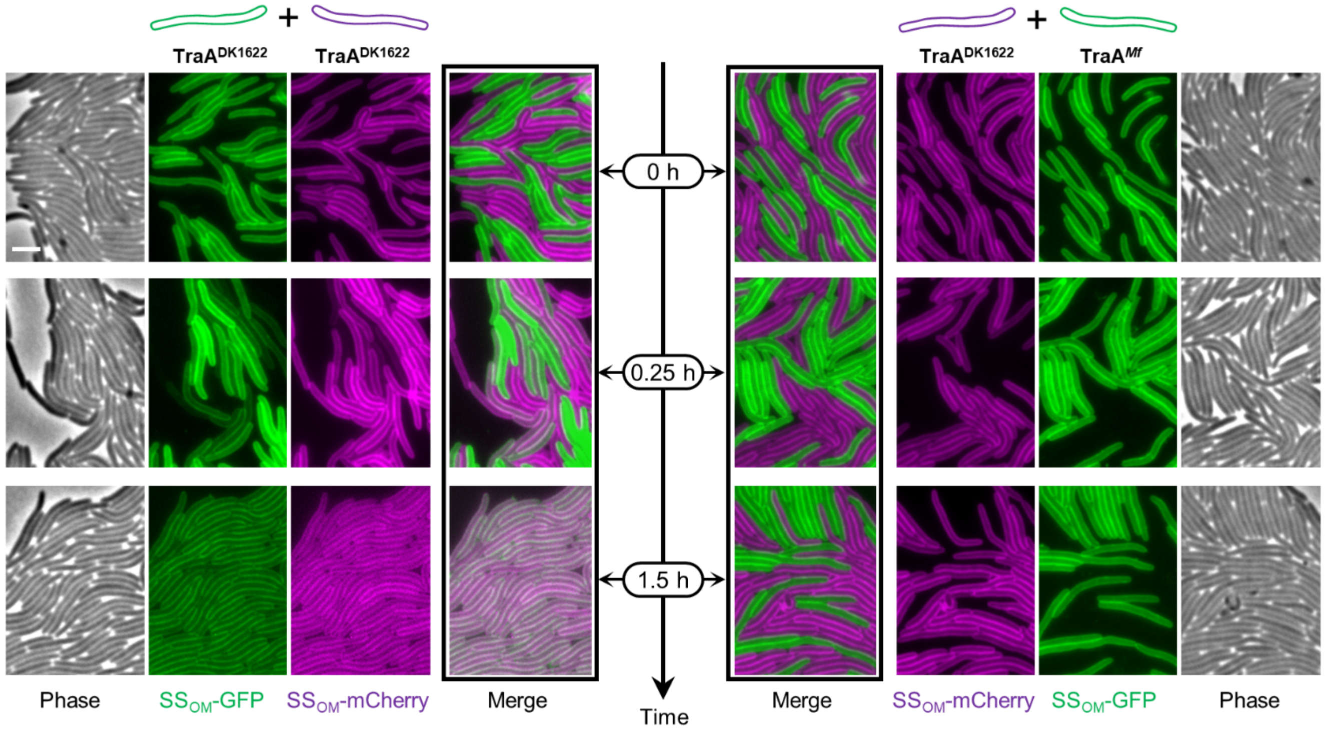

Supplementary Figure 12. TraA recognition and OME transform heterogeneous populations into a homogenous tissue. A complementary figure to **Fig. 5C**, showing a time series of single-channel images as well as merged images. Top cartoons illustrate experimental mixing conditions. Time points after mixing are shown. Scale bar = 1 μm.

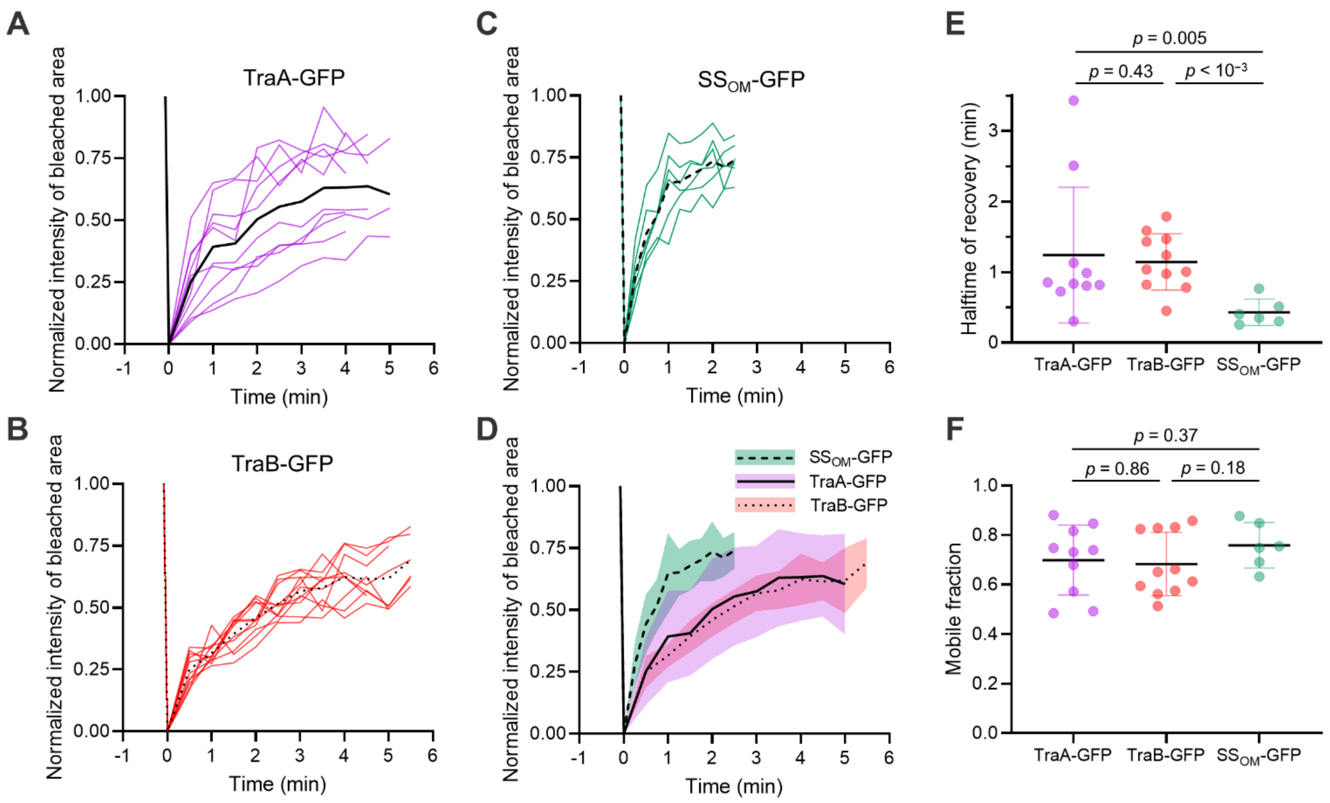

Supplementary Figure 13. FRAP analyses for the OM reporters used in this study. Fluorescence recovery curves of the photobleached regions for TraA-GFP (**A**, *n* = 10 cells), TraB-GFP (**B**, *n* = 11 cells), and SS_{OM}-GFP cells (**C**, *n* = 6 cells). Black lines represent the mean signals. (**D**) Mean signals for TraA-GFP, TraB-GFP, and SS_{OM}-GFP are plotted in the same graph for comparison. The colored bands indicate standard deviations from the means. Halftime of recovery and mobile fraction for different reporters are extracted from FRAP curves and shown in (**E**) and (**F**), respectively. Corresponding mean and standard deviation are shown. *P* values were determined by a two-tailed Mann Whitney test. Source data are provided as a Source Data file.

Supplementary Table 1. Plasmids and strains used in this study

Plasmids	Relevant features	Source
pDP2	*traA* (insertion cassette for creating *traA*::km) in pCR2.1-TOPO, Km^r	[1]
pDP21	P_{pilA}-*traAB* in pSWU19 (Mx8 *attP*), Km^r	[1]
pDP22	P_{pilA} in pSWU19, Km^r	[2]
pDP27	P_{pilA}-*traA* in pSWU19, Km^r	[2]
pDP35	Δ*traAB* cassette in pBJ114, *galK*, Km^r	[3]
pXW6	P_{pilA}-SS_{OM}-*mCherry* in pKSAT, Sm^r	[4]
pXW7	P_{pilA}-*traB* in pCR-XL-TOPO (Δ*neoR/kanR*, Mx9 *attP*), Zeo^r	[5]
pPC4	P_{pilA}-$traAB^{Mf}$ in pSWU19 (Mx8 *attP*), Km^r	[3]
pPC41	P_{pilA}-*traA-gfp-traB* in pSWU19, Km^r	This study
pPC42	P_{pilA}-*traA*(ΔVD)-*gfp-traB* in pSWU19, Km^r	This study
pPC43	P_{pilA}-SS_{OM}-*gfp* in pKSAT, Sm^r	This study
pPC44	P_{pilA}-*traA-mCherry-traB* in pSWU19, Km^r	This study
pPC45	P_{pilA}-$traA^{Mf}$-*gfp-traB* in pSWU19, Km^r	This study
pPC46	P_{pilA}-$traA^{Mf}$-*mCherry-traB* in pSWU19, Km^r	This study
pPC47	P_{pilA}-*traA-gfp* in pSWU19, Km^r	This study
pPC48	P_{pilA}-*traA-gfp* in pCR-XL-TOPO, Zeo^r	This study
pPC49	P_{pilA}-*traA-traB-gfp* in pSWU19, Km^r	This study
pPC50	P_{pilA}-*traB-gfp* in pSWU19, Km^r	This study

Strains	Relevant features	Experimental use	Source
TOP10	*E. coli* cloning strain		Invitrogen
DK1622	Wild-type *M. xanthus,* motile		[6]
DK8601	DK1622 *aglB1* (*aglQ1*) *pilA*::tc, nonmotile, Tc^r	Fig. S6B	[7]
DW1048	DK8601 SS_{IM}-*mCherry*, Km^r, Tc^r	Fig. S6D	[4]
DW1467	DK8601 Δ*traA* (markerless)		[2]
DW1483	DK8601 Δ*traAB* (markerless), Tc^r	Fig. S6C, and 4D	[3]
DW2202	DK8601 (pDP27), Km^r, Tc^r	Fig. S1B	[3]
DW1463	DK8601 (pXW6, pDP21), Km^r, Tc^r, Sm^r	Fig. 1A, and S7	[1]
DK10410	DK1622 Δ*pilA* (markerless)		[7]
DW2270	DK10410 Δ*traAB* (markerless)		This study
DW1466	DK1622 *tgl*::tc Δ*cglC* (markerless), Tc^r	Fig. S6B	[1]

DW2271	DW1483 (pDP21), Km^r, Tc^r	Fig. 3E	This study
DK8615	DK1622 Δ*pilQ* (markerless)		[8]
DW1478	DK8615 (pXW6), Sm^r	Fig. 5C, S1A, and S12	[9]
DW1484	DK1478 *traA*::km, Km^r, Sm^r	Fig. S1A	This study
DW2272	DW1467 (pPC41), Km^r, Tc^r	Fig. 1A, 1C, 2B-C, 3B, 3D, 4A, 5B, S3B, S6E, and S11	This study
DW2273	DW1483 (pPC42), Km^r, Tc^r	Fig. 1A, 1C, and S3B	This study
DW2274	DW2270 (pPC42), Km^r	Fig. S1A	This study
DW2275	DW1483 (pPC43), Tc^r, Sm^r	Fig. S8	This study
DW2276	DW1483 (pDP21, pPC43), Km^r, Tc^r, Sm^r	Fig. 1C, 5A, 5C, S1A, S6C, S6D, S6E, 4D, S9, S10, and S12	This study
DW2277	DW2270 (pPC44), Km^r	Fig. 2A, 3A, 3C, 5A, S1A, S4, S8, and S10B	This study
DW2278	DW2270 (pPC41), Km^r	Fig. 3A, S1A, and S4	This study
DW2279	DW1483 (pPC44), Km^r, Tc^r	Fig. 3B-D, S5, S10A, 5B, and S11	This study
DW2280	DW1483 (pPC45), Km^r, Tc^r	Fig. 3D	This study
DW2281	DW1483 (pPC46), Km^r, Tc^r	Fig. 3D	This study
DW2282	DW2270 (pPC46), Km^r	Fig. S1A	This study
DW2283	DW2281 (pPC48), Km^r, Tc^r, Zeo^r	Fig. 3E	This study
DW2284	DW1483 (pPC47), Km^r, Tc^r	Fig. 4A	This study
DW2285	DW1483 (pPC49), Km^r, Tc^r	Fig. 4A, 4E, S6B, and S6D-E	This study
DW2286	DW1483 (pPC50), Km^r, Tc^r	Fig. 4A, 4C, S7, 4E, and S6C	This study
DW2287	DW1483 (pPC4, pPC43), Km^r, Tc^r, Sm^r	Fig. 5C, and S12	This study
DW2242	DW1467 *traA*^{Mf}*-traB*, Km^r, Tc^r	Fig. 3E, and S7	[3]

Note: *traAB* alleles listed here are from *M. xanthus* DK1622 unless specified.

Supplementary Table 2. Primers used in this study

Primer name	Sequence (5'→3')*
pSWU19-EcoRI-P_{pilA}-F	AGGAAACAGCTATGACCATGATTACGAATTCCGTCATGTTGGACGAGGT
TraA C2-GFP-R	**GCCGCCGCCGCT**GAATGTTTCGGTGTCACAGGC
TraA C2-GFP-F	CACCGAAACATTC**AGCGGCGGCGGCGGC**AGTAAAGGTGAAG
GFP-TraA C3-R	ACTCGACACATGT**GCCGCCGCCGCCGCT**TTTGTAGAGTTCATCCATGCCGT
GFP-TraA C3-F	**GGCGGCGGCGGC**ACATGTGTCGAGTGCAAGAACG
pSWU19-XbaI-HindIII-TraA-R	TGCATGCCTGCAGGTCGACTCTAGAAAGCTTGAAGAGCTGCACGTTGAAG
pSWU19-XbaI-HindIII-TraB-R	TGCATGCCTGCAGGTCGACTCTAGAAAGCTTGGAGTTCTTCACCTCGGACTC
TraA C2-mCherry-F	CACCGAAACATTC**AGCGGCGGCGGCGGC**GCCTCCTCCGAGGACGTC
mCherry-TraA C3-R	ACTCGACACATGT**GCCGCCGCCGCCGCT**CTTGTACAGCTCGTCCATGCC
TraA SS_{OM}-R	GACGATGGGGTCAGTCCCACGGATGCGGAC
TraA VD-F	CATCCGTGGGACTGACCCCATCGTCAAGCTCAAC
pKSAT-HindIII-P_{pilA}-F	ATGAGGATCGTTTCATATGAAGCTTCGTCATGTTGGACGAGGTC
SS_{OM}-GFP-R	TTCACCTTTACTTCTAGAAGGAAACGAGCAACC
SS_{OM}-GFP-F	GTTTCCTTCTAGAAGTAAAGGTGAAGAACTGTTCACCG
pKSAT-EcoRI-GFP-R	TGGATCCCCCGGGCTGCAGGAATTCTTATTTGTAGAGTTCATCCATGCC
$TraA^{Mf}$ C2-GFP-R	**GCCGCCGCCGCT**GTTCGTCTCGAGGTCGCA
$TraA^{Mf}$ C2-GFP-F	CCTCGAGACGAAC**AGCGGCGGCGGCGGC**AGTAAAGGTGAAG
GFP-$TraA^{Mf}$ C3-R	ACTCGACGCAGGT**GCCGCCGCCGCCGCT**TTTGTAGAGTTCATCCATGCCGT
GFP-$TraA^{Mf}$ C3-F	**GGCGGCGGCGGC**ACCTGCGTCGAGTGCAAC
$TraA^{Mf}$ C2-mCherry-F	CCTCGAGACGAACAGCGGCGGCGGCGGCGCCTCCTCCGAGGACGTC
mCherry-$TraA^{Mf}$ C3-R	ACTCGACGCAGGTGCCGCCGCCGCCGCTCTTGTACAGCTCGTCCATGCC

TraB-R	**GCCGCCGCCGCT**CGGCTTCGGAGCCGCGGGGGCCTTCGACTTCGCCTTG
TraB-GFP-F	GGCTCCGAAGCCG**AGCGGCGGCGGCGGC**AGTAAAGGTGAAG
XbaI-TraB-F	GACGACTCTAGAGGATCGACGCCTATATAGACGGGAGATATAATGAAGCCCTCGCACCTG

*Restriction sites are underlined; flexible linkers used for fusing TraA or TraB with FPs are in bold.

Supplementary References

1. Pathak DT, Wei X, Bucuvalas A, Haft DH, Gerloff DL, Wall D. Cell contact-dependent outer membrane exchange in myxobacteria: genetic determinants and mechanism. *PLoS Genetics* **8**, e1002626 (2012).

2. Pathak DT, Wei X, Dey A, Wall D. Molecular recognition by a polymorphic cell surface receptor governs cooperative behaviors in bacteria. *PLoS Genetics* **9**, e1003891 (2013).

3. Cao P, Wall D. Self-identity reprogrammed by a single residue switch in a cell surface receptor of a social bacterium. *Proceedings of the National Academy of Sciences* **114**, 3732-3737 (2017).

4. Wei X, Pathak DT, Wall D. Heterologous protein transfer within structured myxobacteria biofilms. *Molecular Microbiology* **81**, 315-326 (2011).

5. Cao P, Wei X, Awal RP, Müller R, Wall D. A Highly Polymorphic Receptor Governs Many Distinct Self-Recognition Types within the *Myxococcales* Order. *mBio* **10**, e02751-02718 (2019).

6. Dey A, Vassallo CN, Conklin AC, Pathak DT, Troselj V, Wall D. Sibling rivalry in *Myxococcus xanthus* is mediated by kin recognition and a polyploid prophage. *Journal of Bacteriology* **198**, 994-1004 (2016).

7. Wall D, Kaiser D. Alignment enhances the cell-to-cell transfer of pilus phenotype. *Proceedings of the National Academy of Sciences* **95**, 3054-3058 (1998).

8. Wall D, Kolenbrander PE, Kaiser D. The *Myxococcus xanthus pilQ* (*sglA*) gene encodes a secretin homolog required for type IV pilus biogenesis, social motility, and development. *Journal of Bacteriology* **181**, 24-33 (1999).

9. Dey A, Wall D. A genetic screen in *Myxococcus xanthus* identifies mutants that uncouple outer membrane exchange from a downstream cellular response. *Journal of Bacteriology* **196**, 4324-4332 (2014).
