# Supplementary material for: Direct visualization of a molecular handshake that governs kin recognition and tissue formation in myxobacteria
Source: Nat Commun. 2019 Jul 12;10:3073. doi: 10.1038/s41467-019-11108-w (PMC6626042; doi:10.1038/s41467-019-11108-w)
Supplement: Supplementary file 3 — Description of Additional Supplementary Files [file 41467_2019_11108_MOESM3_ESM.pdf]

Description of Additional Supplementary Files

File Name: Supplementary Movie 1
Description: **Interaction between two cells bearing TraA-mCherry.** Time interval between frames was 30 sec. The corresponding image series are shown in Fig. 2A.

File Name: Supplementary Movie 2
Description: **Transfer of SSOM-GFP between two kin cells**. The donor cell (right) and the recipient cell (left) harbor compatible TraA. Time interval between frames was 45 sec. Corresponding images are shown in Fig. 5A.

File Name: Supplementary Movie 3
Description: **A working model of how a fluid cell surface receptor recognizes and assembles kin cells into a tissue.**
